# Prevalence and Characteristics of Inspiration-Induced Negative Left Atrial Pressure during Pulmonary Vein Isolation

**DOI:** 10.3390/jcdd10030101

**Published:** 2023-02-26

**Authors:** Takenori Ikoma, Yoshihisa Naruse, Yutaro Kaneko, Tomoaki Sakakibara, Taro Narumi, Makoto Sano, Satoshi Mogi, Kenichiro Suwa, Hayato Ohtani, Masao Saotome, Tsuyoshi Urushida, Yuichiro Maekawa

**Affiliations:** Division of Cardiology, Internal Medicine III, Hamamatsu University School of Medicine, 1-20-1 Handayama, Higashi Ward, Hamamatsu 431-3192, Japan

**Keywords:** atrial fibrillation, catheter ablation, air embolism, inspiration-induced negative left atrial pressure

## Abstract

Background: Atrial fibrillation (AF) ablation is performed under deep sedation, which may cause inspiration-induced negative left atrial pressure (INLAP) associated with deep inspiration. INLAP could be the cause of periprocedural complications. Methods: We retrospectively enrolled 381 patients with AF (mean age, 63.9 ± 10.8 years; 76 women; 216 cases of paroxysmal AF) who underwent CA under deep sedation using an adaptive servo ventilator (ASV). Patients whose LAP was not obtained were excluded. INLAP was defined as <0 mmHg of mean LAP during inspiration immediately after the transseptal puncture. The primary and secondary endpoints were the presence of INLAP and the incidence of periprocedural complications. Results: Among 381 patients, INLAP was observed in 133 (34.9%). Patients with INLAP had higher CHA_2_DS_2_-Vasc scores (2.3 ± 1.5 vs. 2.1 ± 1.6) and 3% oxygen desaturation indexes (median 18.6 (interquartile range 11.2–31.1) vs. 15.7 (8.1–25.3)), and higher prevalence of diabetes mellitus (23.3 vs. 13.3%) than patients without INLAP. Air embolism occurred in four patients with INLAP (3.0 vs. 0.0%). Conclusion: INLAP is not rare in patients undergoing CA for AF under deep sedation with ASV. Much attention should be paid to the possibility of air embolism in patients with INLAP.

## 1. Introduction

The incidence and prevalence of atrial fibrillation (AF) have increased in recent years. AF is associated with an increased risk of cerebrovascular accidents, heart failure, and all-cause death [1]. Early rhythm control therapy has been reported to improve the prognosis of patients with AF [2]. Additionally, rhythm control therapy with catheter ablation (CA) can improve a patient’s quality of life [3]. Currently, CA is recognized as a reasonable therapeutic option for AF [4].

**Figure 1:**
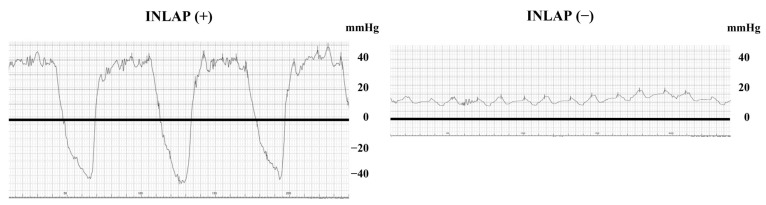
Representative LAP curve during inspiration with and without INLAP. The bold line indicates a level of 0 mmHg. The left panel shows a representative case with INLAP. LAP shows a significant respiratory change from −30 to 40 mmHg. Abbreviations: INLAP, inspiration-induced negative left atrial pressure; LAP, left atrial pressure.

CA for AF is effective; however, the complication rate of CA in the acute phase is 7.48% [5]. Among the complications, air embolism is a crucial complication that can cause acute systemic embolism [6]. The most common embolisms are coronary air embolisms, with a prevalence of 2.6% in cryoballoon ablations [7]. Negative left atrial pressure (LAP), in conjunction with air-leaking sheaths, is essential for air intrusion into the left atrium (LA) [8]. In particular, chest wall expansion and diaphragm descent due to deep inspiration with airway obstruction induce negative atrial pressure [9]. However, the prevalence and clinical characteristics of inspiration-induced negative LAP (INLAP; Figure 1) in patients undergoing CA for AF are unknown. In this study, we aimed to evaluate the prevalence and clinical impact of INLAP during CA for AF.

## 2. Materials and Methods

### 2.1. Participants

**Figure 2:**
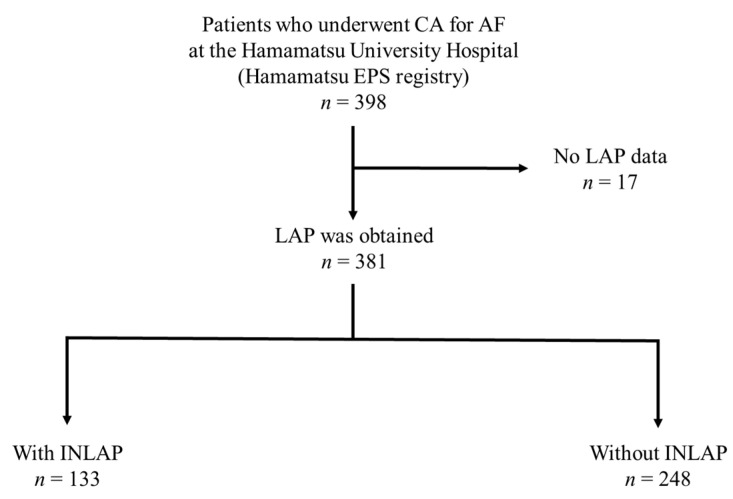
Schematic diagram for patient selection in the study. Abbreviations: CA, catheter ablation; AF, atrial fibrillation; LAP, left atrium pressure; INLAP, inspiration-induced negative left atrium pressure.

This study is a registry-based study and the registry itself prospectively collected the data. We retrospectively enrolled 398 Japanese patients who underwent CA for AF at the Hamamatsu University Hospital between March 2017 and March 2020, using a prospectively collected ablation database (Hamamatsu EPS registry). Patients aged 18 years or older were eligible. Seventeen patients whose LAP was not measured during CA were excluded from this study (Figure 2). The primary endpoint was the presence of INLAP measured immediately after a transseptal puncture, and the secondary endpoint was the incidence of periprocedural complications. Patients were divided into two groups according to the presence or absence of INLAP, and the procedural parameters were compared between the groups. Data on age, sex, body mass index, comorbidities, and medications were collected.

Paroxysmal (terminated within 7 days) and persistent AF (lasting > 7 days) were defined according to the current guidelines [1]. Before ablation, patients underwent a blood examination, transthoracic echocardiography, and electrocardiogram-gated contrast-enhanced computed tomography (CT) scanning. The LA volume index was obtained by dividing the LA volume by body surface area (BSA). BSA was calculated by the following formula: BSA = (height)^0.725^ ×(body weight)^0.425^ × 0.007184. Overnight pulse oximetry was performed 1 day after CA for AF. Written informed consent was obtained from all the patients before enrollment. The protocol was performed according to the Declaration of Helsinki and approved by the Human Investigations Committee of the Hamamatsu University School of Medicine (approval number #20-361).

### 2.2. Measurement of Intracardiac Pressure and the Definition of INLAP and Inspiration-Induced Negative Right Atrial Pressure

Inferior vena cava pressure, right atrial pressure (RAP), and LAP were measured under sedation and adaptive servo-ventilator (ASV) support without the use of the nasal airway by connecting a pressure transducer with a T-shaped stopcock of the SL0^TM^ 8.5 Fr Swartz Braided Sheath (Abbott, St. Paul, MN, USA). These pressures were measured at an average of four to five respiratory cycles. RAP and LAP were measured immediately after the sheath’s tip reached the right atrium (RA) and LA. INLAP or inspiration-induced negative right atrial pressure (INRAP) was defined as a mean LAP or RAP < 0 mmHg during inspiration. Representative cases of patients with and without INLAP are shown in Figure 1. The pressure data during the procedure was obtained by an electrophysiological specialist independent of this study.

### 2.3. Electrophysiological Study and CA

Rivaroxaban, apixaban, and edoxaban treatments were omitted on the morning of the ablation procedure; however, dabigatran and warfarin treatments were uninterrupted. Antiarrhythmic drugs prescribed before the ablation were continued. The procedure was performed under intravenous sedation with midazolam (0.1 mg/kg/h) and dexmedetomidine (0.2 µg/kg/h following 10 min of bolus infusion of 4 µg/kg/h). The sedation level was monitored using the Richmond Agitation–Sedation Scale (RASS) and a bispectral index (BIS). The infusion rate of sedatives (midazolam and dexmedetomidine) was adjusted to maintain a BIS level between 50 and 80. Respiration was supported by ASV under the uniform settings (S/T mode, inspiratory positive airway pressure at 8 cm H_2_O, expiratory positive airway pressure at 4 cm H_2_O, fraction of inspired oxygen (FiO_2_) 40%, and respirate 15 cycles/min) at the beginning of the procedure. Confirming mask fitting, changing mask size or raising the oxygen concentration level were offered according to the operator’s judgement when there were problems with oxygenation or other problems. Intravenous heparin was administered to maintain an activated clotting time of 300–400 s during the procedure. 

Electroanatomical mapping was performed in sinus rhythm or atrial pacing, and electrical cardioversion was performed when the patient presented with AF during voltage mapping. Electroanatomical voltage maps of the LA were created using a 20-pole circular mapping catheter (Optima or Advisor-VL, Abbott, St. Paul, MN, or LASSO, Biosense Webster, Diamond Bar, CA, USA) or a multi-electrode high-density mapping catheter (HD grid, Abbott, or PENTARAY, Biosense Webster), with three-dimensional electroanatomical mapping systems (Navx-Ensite Velocity, Abbott, or CARTO 3, Biosense Webster). In both mapping systems, low voltage zones (LVZs) were defined as sites with a peak-to-peak electrogram amplitude of < 0.50 mV. Pulmonary vein isolation (PVI) was performed in all patients with de novo AF procedures using open-irrigated radiofrequency catheters, cryoballoons (Arctic Front Advance, Medtronic, Minneapolis, MN, USA), or laser balloons (HeartLight, CardioFocus, Marlborough, MA, USA). A water bucket developed to prevent air intrusion (AirTray, NISSHO, Shizuoka, Japan) was used in balloon catheter insertion. PVI was confirmed in patients with repeated procedures, and the pulmonary vein (PV) was re-isolated if PV reconnection was observed. Additional ablation, consisting of LA posterior box isolation [10], superior vena cava isolation, or ablation of the spatiotemporal dispersion area [11], was performed at the operator’s discretion. Air embolisms during CA for AF were defined as coronary air embolisms with ST segment elevation in inferior leads confirmed with coronary angiography or air intrusion images (e.g., in the LA or aorta).

### 2.4. Statistical Analysis

Continuous variables are expressed as mean ± standard deviation or median (interquartile range (IQR)). Between-group comparisons were performed using an unpaired *t*-test or Mann–Whitney U test. All categorical variables were presented as numbers and percentages for each group and were compared using the chi-square test or Fisher’s exact test. Logistic regression analysis was performed to detect any independent, significant predictors by adjusting the variables with multivariable models (reported as odds ratios (ORs) with 95% confidence intervals (Cis)). Variables that achieved statistical significance (*p* < 0.05) or were close to significance (*p* < 0.1) in the Spearman’s rank correlation coefficient and possible factors were included in the multiple linear regression analysis. Correlations between the INLAP and INRAP scores were analyzed using Spearman’s correlation coefficient, and freedom from AF recurrence was assessed using the Kaplan–Meier method. Survival curves were compared between the groups using a log-rank test. To evaluate the predictive value of the mean RAP during inspiration for INLAP, a receiver operating characteristic analysis was performed, calculating the area under the curve and evaluating possible cutoff points. Statistical significance was defined as *p* < 0.05. All statistical analyses were performed using SPSS version 26.0 (IBM, Armonk, NY, USA). Graphs were compiled using Prism 7.03 (GraphPad, La Jolla, CA, USA).

## 3. Results

### 3.1. Subject Characteristics

A total of 381 patients (76 (19.9%) women; mean age, 63.9 ± 10.8 years) were analyzed. Table 1 presents the patients’ baseline characteristics. There were 216 (56.7%) patients with paroxysmal AF. A history of PVI was present in 40 patients (10.5%). The mean LA diameters assessed by echocardiography and the LA volume index by cardiac CT were 39.1 mm and 71.5 mL/m^2^, respectively.

### 3.2. Feature of Patients with INLAP

**Figure 3:**
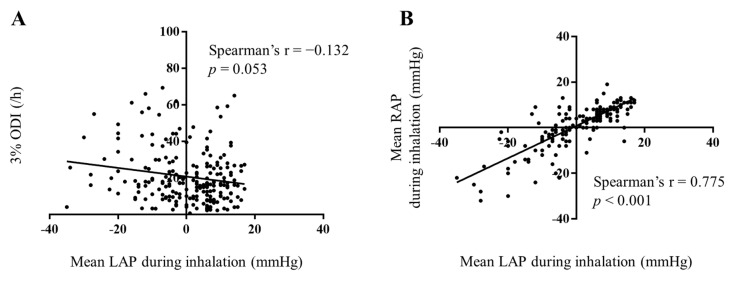
Association between the mean LAP during inspiration and 3% ODI value (**A**) or mean RAP during inspiration (**B**). The correlation is significant between mean LAP and mean RAP during inspiration (r = 0.775). Abbreviations: LAP, left atrial pressure; ODI, oxygen desaturation index; RAP, right atrial pressure.

Within the entire cohort, 133 patients (34.9%) had INLAP. Patients with INLAP had a higher prevalence of diabetes mellitus (*p* = 0.013) than those without INLAP. Higher CHA_2_DS_2_-Vasc scores (*p* = 0.043) and 3% oxygen desaturation indexes (ODIs; *p* = 0.009) were observed in patients with INLAP. However, 3% ODI correlated very weakly with the mean LAP during inspiration (Figure 3A). There were no significant between-group differences regarding medications and the results of laboratory testing, echocardiography, and CT findings (Table 1).

No significant differences were observed in inferior vena cava (IVC) pressure during expiration; however, IVC (*p* < 0.001), RA (*p* < 0.001), and LA pressure (*p* < 0.001) during inspiration were lower in patients with INLAP. A higher prevalence of INRAP was detected in patients with INLAP (*p* < 0.001). Moreover, the mean RAP during inspiration correlated well with the mean LAP during inspiration (Figure 3B). Additional ablation for the cavotricuspid isthmus line and mitral isthmus was conducted less frequently in patients with INLAP (*p* = 0.007 and 0.040, respectively). Air embolism occurred in four patients with INLAP. Air embolism was suspected from ST segment elevation in the inferior leads of an electrocardiogram, fall in blood pressure, or bradycardia. Air embolism was observed after inserting the 20-pole circular mapping catheter into the LA of two patients, a multi-electrode, high-density mapping catheter into the LA of one patient, and cryoballoon into the LA of one patient. Emergent coronary angiography showed an air embolism in the right coronary artery. Fortunately, proper treatment, such as air aspiration via a catheter, ensured that there was no residual damage, including focal symptoms of stroke and myocardial wall motion impairment, in any of the cases. In contrast, no patients without INLAP experienced air embolism during CA for AF (*p* = 0.014). Median LA pressure during the inspiration period did not differ significantly between INLAP patients with or without air embolism (−9.5 (−4.5–−13.8) mmHg vs. −8.0 (−3.5–−14.0) mmHg; *p* = 0.0812). The incidence of cardiac tamponade tended to be higher in patients with INLAP than in those without (*p* = 0.053). The RASS and BIS index did not differ between patients with and without INLAP. No significant between-group difference was observed in the type of ablation technology and procedural and fluoroscopic times (Table 2).

**Figure 4:**
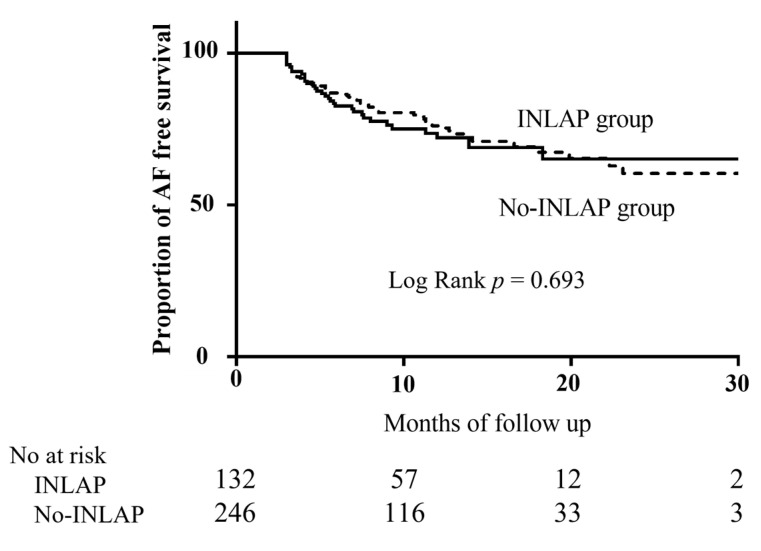
Kaplan–Meier curve of freedom from AF recurrence. Abbreviations: AF, atrial fibrillation; INLAP, inspiration-induced negative left atrial pressure.

During a median follow-up period of 8.7 (IQR 6.16–13.3) months, AF recurrence occurred in 92 (24.1%) patients. The Kaplan–Meier survival curve showed no significant difference in AF recurrence between patients with and without INLAP (*p* = 0.693 by log-rank test; Figure 4).

### 3.3. Prediction of INLAP

Multiple linear regression analysis was performed to identify the factors associated with the decrease in LA pressure in the inspiration period. Age, body mass index, diabetes mellitus, INRAP, 3% ODI (categorized into the following three groups according to their value: <5, 5–10, and >10), and history of prior PVI were included as factors. Multiple linear regression analysis demonstrated that the presence of INRAP was independently associated with the decrease in LA pressure in the inspiration period (*p* < 0.001; Table 3). 

**Figure 5:**
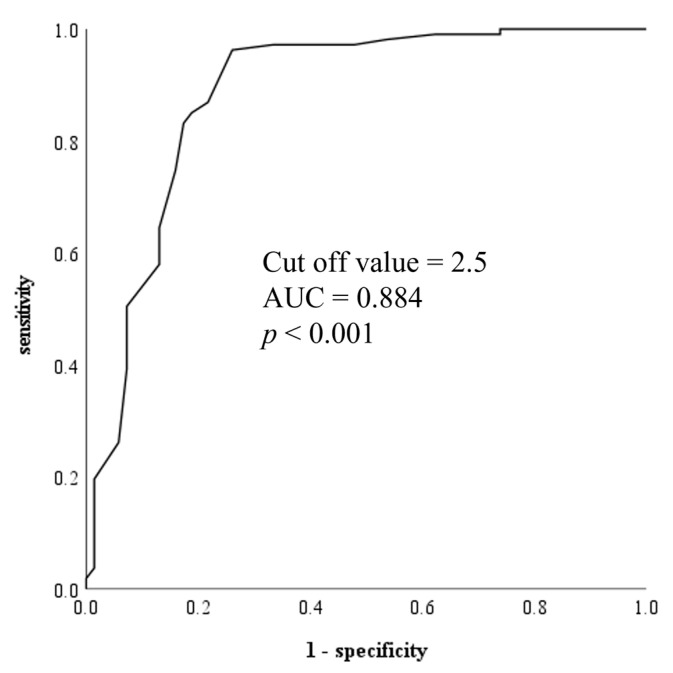
Receiver operating characteristic curve of RAP during inspiration. Abbreviations: AUC, area under the curve; RAP, right arterial pressure.

The receiver operating characteristic curve used to evaluate the predictive value of the mean RAP during inspiration in distinguishing between those with and without INLAP during PVI revealed an area under the curve of 0.884 (*p* < 0.001). An RAP during inspiration that was less than 2.5 mmHg identified the presence of INLAP with a sensitivity and specificity of 83.2% and 82.6%, respectively (Figure 5).

## 4. Discussion

### 4.1. Main Findings

The main findings of this study were as follows: 1) approximately one third of the patients undergoing PVI under deep sedation with ASV had INLAP during the ablation procedure, 2) the presence of INLAP can be predicted from the RAP, 3) the presence of INLAP could cause air embolisms during PVI, and 4) the presence of INLAP was not associated with AF recurrence after PVI.

### 4.2. Clinical Impact and Importance of Air Embolism

The Japanese Heart Rhythm Society emphasized the risk of air embolism during cryoballoon ablation in 2018. In addition, a comprehensive review suggested the importance of detecting and managing air embolism [12]. An ex vivo study revealed that air intrusion, the cause of air embolism, is associated with the following two factors: the catheter system (comprising catheters of different vascular diameters) and the negative pressure in the sheath [13]. It was reported that negative LAP was an important factor in air embolism [8], and ex vivo experiments that evaluated air-tightness in response to negative pressures revealed air aspiration at −11 to −13 mmHg of suction [13]. All four cases of air embolism in this study were observed in patients with INLAP. Air intrusion in the left heart system can lead to more severe conditions or after-effects (i.e., myocardial or brain infarctions) than that of the right heart system (pulmonary embolism). Therefore, much attention should be paid to air embolisms in patients with INLAP.

### 4.3. Possible Mechanisms of INLAP

In this study, INLAP was observed in 133 (34.9%) patients under deep sedation with ASV, signifying that INLAP is not rare and could even occur with ASV in patients undergoing PVI. Patients with obstructive sleep apnea (OSA) show negative LA pressure [13]. Although the 3% ODI values were higher in patients with INLAP, the correlation was weak. In addition, this study revealed that INLAP was not associated with LA volume and the presence of LVZs, which indicates LA remodeling [14].

Furthermore, the presence of INLAP did not affect the recurrence of AF after PVI. From the above, it seems that INLAP is not associated with AF’s pathophysiology or PVI’s effectiveness. Additionally, since OSA was associated with the presence of LA enlargement [15] and recurrence of AF after CA [16], the severity of OSA could not be associated with the presence of INLAP.

A previous study that reports LAP during atrial septal defect/patent foramen ovale closure revealed that sedation provoked a marked decline in the mean inspiratory LAP compared to non-sedated patients [8]. Deep sedation using propofol or midazolam is associated with a profound drop in LAP [17,18]. In addition, a previous report revealed that patients that require airway management tools due to airway obstruction during CA showed substantial negative esophageal pressure compared to those without airway obstruction [19]. Since our findings showed no significant differences in IVC pressure during expiration, the patients’ circulating plasma volume statuses, including overflow and dehydration, did not affect the presence of INLAP. Thus, airway obstruction due to the decrease in muscle tonus under deep sedation, which could not be removed using ASV, could cause INLAP. 

A recent report showed that the prevalence of INLAP significantly reduced after ASV (pre-ASV 73% vs. post-ASV 14%) [20], which is lower than our result of 35%. A possible reason for the difference in the prevalence of INLAP in patients during PVI with ASV between the two studies could be that the BIS level was higher in the previous report than in the present study (68.5 ± 12.7 vs. 59.3 ± 14.4). This finding that deeper sedation was associated with a higher prevalence of INLAP could support this as a suspected mechanism of INLAP rather than the severity of sleep apnea. Deep sedation was mainly responsible for INLAP development in patients undergoing PVI under deep sedation with ASV.

### 4.4. Prevention of Complications Due to INLAP

We found that RAP could predict the decrease in LA pressure during the inspiration period, which is a crucial finding. Predicting the presence of INLAP and taking proper provisions before a transseptal puncture enables us to prevent systemic air embolisms. The RAP can be measured easily by connecting a long sheath to a pressure transducer. We should pay great attention to the occurrence of systemic air embolisms if a mean RAP during inspiration < 2.5 mmHg is observed before a transseptal puncture. It has been reported that a water bucket developed to prevent air intrusion (AirTray, NISSHO, Shizuoka, Japan or SAFE BOAT, DVx, Tokyo, Japan) could reduce the incidence of air embolisms [7]. Such equipment should be used for patients with INLAP in the insertion of any catheters, as we reported air embolism even when a catheter other than a balloon catheter was inserted. Furthermore, since the incidence of INLAP was high (34.9% in this study) under deep sedation with ASV, the routine use of such devices may be recommended. Furthermore, our findings demonstrated that patients with INLAP have an increased risk of cardiac tamponade than those without INLAP. An unstable respiration pattern is often observed in patients with INLAP due to airway obstruction, which could be responsible for the occurrence of cardiac tamponade because of the sudden increase in the contact force of an ablation catheter.

Additionally, we hypothesize that the use of the nasal airway in addition to ASV could help to relieve airway obstruction in patients with INLAP under deep sedation with ASV and we are conducting further research to assess this hypothesis.

### 4.5. Study Limitations

This study had several limitations. First, this was a single-center retrospective study, and the small sample size limited its predictive power. Second, four cases of air embolism were observed in this study. Although the CA procedures were performed by well-trained operators, the incidence of air embolisms and cardiac tamponade may still be affected by the skill of the operators. In addition, it was reported that the asymptomatic cerebral embolism during cryoballoon ablation of AF was observed in 22.9% of patients [21]. Since brain magnetic resonance imaging was not performed routinely after ablation, silent air embolisms were not evaluated. In addition, as the coronary angiography was conducted for patients with ST elevation in ECG, slight changes that are difficult to recognize, including transient ST elevation, could be underestimated. Third, since all the patients received CA procedures under deep sedation using an ASV, we have no control group without sedation and the use of ASV. Fourth, we did not evaluate the detailed cardiac morphology and hemodynamics of the patients, including interventricular septum thickness, trans mitral flow velocity pattern, and blood pressure that could affect the presence of INLAP. Although we did not find any relationship between INLAP and BNP levels in the present study, there is a report in the literature that discusses the relationship between BNP and left atrial morphology [22]. Further investigation that explores the relationships among LA morphology, biomarkers, and the presence of INLAP is needed.

## 5. Conclusions

INLAP during CA was not rare in patients undergoing CA for AF under deep sedation, even with ASV. Much attention should be paid to air embolisms in patients with INLAP.

## Figures and Tables

**Table 1:** Demographic and baseline characteristics in patients with and without inspiration-induced negative left atrial pressure.

	All (*n* = 381)	INLAP (*n* = 133)	No INLAP (*n* = 248)	*p*-Value
Demographics				
Age, years	63.9 ± 10.8	65.2 ± 9.3	63.2 ± 11.5	0.077
Female, *n* (%)	76 (19.9)	24 (18.0)	52 (21.0)	0.496
Body mass index, kg/m^2^	24.6 ± 4.1	25.0 ± 4.4	24.4 ± 4.0	0.173
Current smoker, *n* (%)	41 (12.0)	17 (13.8)	24 (11.0)	0.443
Use of alcohol, *n* (%)	194 (56.6)	72 (58.5)	122 (55.5)	0.581
Paroxysmal AF, *n* (%)	216 (56.7)	77 (57.9)	139 (56.0)	0.729
History of prior PVI, *n* (%)	40 (10.5)	5 (3.8)	35 (14.1)	0.002
Clinical characteristics				
HT, *n* (%)	198 (52.0)	75 (56.4)	123 (49.6)	0.206
Dyslipidemia, *n* (%)	98 (25.7)	36 (27.1)	62 (25.0)	0.660
DM, *n* (%)	64 (16.8)	31 (23.3)	33 (13.3)	0.013
Stroke, *n* (%)	38 (10.0)	16 (12.0)	22 (8.9)	0.327
Heart failure, *n* (%)	101 (26.5)	36 (27.1)	65 (26.2)	0.856
3% ODI	17.0 (9.5–26.7)	18.6 (11.2–31.1)	15.7 (8.1–25.3)	0.009
CHADS_2_ score, points	1.3 ± 1.2	1.5 ± 1.2	1.3 ± 1.2	0.071
CHA_2_DS_2_-Vasc score, points	2.2 ± 1.5	2.3 ± 1.5	2.1 ± 1.6	0.043
Medications				
ACE-Is / ARBs, *n* (%)	137 (36.0)	54 (40.6)	83 (33.5)	0.167
Beta-blockers, *n* (%)	225 (59.1)	83 (62.4)	142 (57.3)	0.330
Type I AADs, *n* (%)	153 (40.2)	45 (33.8)	108 (43.5)	0.065
Type III AADs, *n* (%)	31 (8.1)	9 (6.8)	22 (8.9)	0.474
DOAC, *n* (%)	350 (88.5)	126 (94.7)	224 (90.3)	0.133
Laboratory data				
Hb, g/dL	14.2 ± 1.6	14.2 ± 1.6	14.2 ± 1.6	0.957
eGFR, ml/min/1.73 m^2^	63.7 ± 16.9	62.7 ± 13.8	64.2 ± 18.3	0.462
NT-proBNP, pg/ml	243.0 (72.0–610.0)	240.0 (82.0–683.0)	249.0 (70.0–575.0)	0.409
Echocardiography				
LVEF, %	62.7 ± 10.2	62.4 ± 11.0	62.8 ± 9.9	0.765
LAD, mm	39.1 ± 7.1	39.8 ± 7.3	38.7 ± 7.0	0.159
LVDd, mm	47.7 ± 5.9	48.0 ± 5.9	47.5 ± 5.9	0.434
LVDs, mm	31.3 ± 6.3	31.5 ± 6.6	31.2 ± 6.2	0.683
Computed tomography				
LA volume index, ml/m^2^	71.5 ± 39.1	70.6 ± 24.2	72.1 ± 45.1	0.727

Data are presented as mean ± SD, median (IQR), or number (%). Abbreviations: AADs, antiarrhythmic drugs; ACE-Is, angiotensin-converting enzyme inhibitors; AF, atrial fibrillation; ARBs, angiotensin receptor blockers; DM, diabetes mellitus; DOAC, direct oral anticoagulant; eGFR, estimated glomerular filtration rate; Hb, hemoglobin; HT, hypertension; INLAP, inspiration-induced negative left atrial pressure; IQR, interquartile range; LA, left atrium; LAD, left atrial diameter; LVDd, left ventricular end-diastolic diameter; LVDs, left ventricular end-systolic diameter; LVEF, left ventricular ejection fraction; NT-proBNP, N-terminal prohormone B-type natriuretic peptide; ODI, oxygen desaturation index; PVI, pulmonary vein isolation; SD, standard deviation.

**Table 2:** Procedural characteristics in patients with and without INLAP.

	All (*n* = 381)	INLAP (*n* = 133)	No INLAP (*n* = 248)	*p*-Value
INRAP, *n* (%)	55 (31.2)	51 (73.9)	4 (3.7)	< 0.001
RA pressure				
Inspiration period, mmHg	1.2 ± 9.3	−6.2 ± 9.9	6.1 ± 4.5	< 0.001
Expiration period, mmHg	11.4 ± 5.6	12.0 ± 7.0	11.1 ± 4.5	0.273
LA pressure				
Inspiration period, mmHg	1.5 ± 10.4	−10.1 ± 8.0	7.8 ± 4.4	< 0.001
Expiration period, mmHg	15.9 ± 6.5	17.9 ± 7.3	14.9± 5.8	< 0.001
IVC pressure				
Inspiration period, mmHg	5.8 ± 5.8	2.8 ± 6.8	7.9 ± 3.9	< 0.001
Expiration period, mmHg	11.2 ± 6.0	11.5 ± 7.7	11.0 ± 4.5	0.539
Low voltage zones, *n* (%)	77 (20.2)	33 (24.8)	44 (17.7)	0.101
Type of ablation technology				
Radiofrequency catheters, *n* (%)	106 (27.8)	43 (32.3)	63 (25.4)	0.150
Cryoballoons and laser balloons, *n* (%)	275 (72.2)	90 (67.7)	185 (74.6)	0.150
Additional ablation, *n* (%)				
CTI, *n* (%)	179 (47.0)	50 (37.6)	129 (52.0)	0.007
Bottom line, *n* (%)	123 (32.3)	39 (29.3)	84 (33.9)	0.365
Mitral isthmus, *n* (%)	25 (6.6)	4 (3.0)	21 (8.5)	0.040
Complications				
Air embolism, *n* (%)	4 (1.0)	4 (3.0)	0 (0.0)	0.014
Diaphragmatic paralysis, *n* (%)	9 (2.4)	4 (3.1)	5 (2.0)	0.725
Bleeding from puncture site, *n* (%)	6 (1.6)	2 (1.5)	4 (1.6)	1.000
Cardiac tamponade, *n* (%)	7 (1.8)	5 (3.8)	2 (0.8)	0.053
Sedation level				
RASS	−4.0 (−4.0–−5.0)	−4.0 (−4.0–−5.0)	−4.0 (−4.0–−4.0)	0.131
BIS	57.0 (48.0–70.0)	58.5 (50.8–73.0)	56.0 (47.0–68.0)	0.098
Procedure time, minute	207.3 ± 83.8	208.5 ± 81.2	206.6 ± 85.3	0.834
Fluoroscopic time, minute	53.4 ± 34.8	50.5 ± 32.4	54.9 ± 35.9	0.234

Data are presented as mean ± SD, median (IQR), or number (%). Abbreviations: BIS, bispectral index; CBs, cryoballoons; CTI, cavotricuspid isthmus line; INLAP, inspiration-induced negative left atrial pressure; INRAP, inspiration-induced negative right atrial pressure; IQR, interquartile range; IVC, inferior vena cava; RASS, Richmond Agitation–Sedation Scale; SD, standard deviation.

**Table 3:** Multiple linear regression analysis of variables associated with predictors of the decrease in LA pressure during the inspiration period (presence of INRAP predicts the decrease in LA pressure during inspiration period).

Variable	Beta	Standard Error	T Value	*p*-Value
(Constant)	1.785	6.358	0.281	0.779
Age	0.002	0.062	0.034	0.973
BMI	0.201	0.158	1.267	0.207
DM	0.954	1.524	0.626	0.532
INRAP	−16.795	1.238	−13.571	< 0.001
3% ODI	−0.780	0.876	−0.890	0.375
History of prior PVI	0.063	2.225	0.028	0.978
R^2^ = 0.532 (*p* < 0.001)				

The 3% ODI is categorized into the following three groups according to its value: <5, 5–10, and >10. Abbreviations: BMI, body mass index; CI, confidence interval; DM, diabetes mellitus; INLAP, inspiration-induced negative left atrial pressure; INRAP, inspiration-induced negative right atrial pressure; ODI, oxygen desaturation index; PVI, pulmonary vein isolation.

## Data Availability

The deidentified participant data will not be shared.
